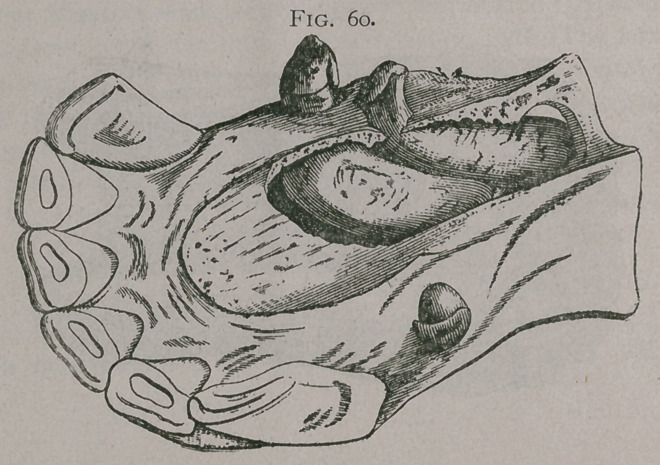# Age of the Horse, Ox, Dog, and Other Domesticated Animals

**Published:** 1891-03

**Authors:** R. S. Huidekoper

**Affiliations:** Veterinarian


### AGE OF THE HORSE, OX, DOG, AND OTHER DOMESTICATED ANIMALS.

By R. S. Huidekoper, M.D., Veterinarian.

[*Continued from page 83.*]

### Irregularities of the Dental System.

The dental system offers a large number of very interesting and important irregularities. When these exist the horse is said to have an irregular mouth, or to be falsely marked. Some of the irregularities are without importance, while others may influence the wearing of the teeth to such a degree as to make the determination of age somewhat difficult, except by a careful study of all the changes.

The irregularities can be classified as follows:

1st, Number—augmentation and diminution. 2nd, Form of the incisors. 3rd, Uniting of two incisors. 4th, The form of the cup, fissure of the dental cups. 5th, Depth of the dental cup and size of its cavity (begue). 6th, Fault of length, or excess of size of one of the jaws, prognathism, brachy-gnathism, excess of length of the superior incisive arch. 7th, Excess or fault of use. 8th, Marks produced by cribing. 9th, Fraudulent alterations, removal of the milk teeth, bishoping, filing the corners.

### Irregularities in Number.—Augmentation.

*Incisors*:—The most curious example of this anomaly is one which was noted for the first time by Lafosse, 1772. He states that he found horses with the double rows of incisive teeth. This anomaly was again noted by Goubaux, in 1842. His case had two rows of incisors of second dentition in each jaw, making twenty-four in all.

Augmentation or duplication of the incisors of second dentition for one or two pairs of teeth are not rare. The accompanying plate gives an excellent example. In figure 56, there are two

FIG. 56.

FIG. 57.

supernumerary pinchers *aa*, and one supernumerary intermediate tooth *b*. Figure 57, shows two supernumerary intermediate teeth *a* and *b*. Figure 58, has an intermediate tooth *a*, directed transversely and held in place below the incisive foramen by a bony bridge.

FIG. 58.

Figure 59, shows a pincher tooth similarly deflected. All these teeth are of second dentition.

FIG. 59.

FIG. 59 *a*.

Supernumerary teeth of the lower jaw are less common than in the upper one; they sometimes however, occur. The presence of supernumerary teeth rarely modifies the wearing of the others in the marking of age. Supernumerary teeth are usually firmly held in their alveolar cavities and rarely alter the shape of the arch of the incisors, except when milk teeth are still remaining, with which they should not be confounded.

*Tush Teeth:*—Goubaux reports a case of an ass with supernumerary tush teeth which seems unique, Fig. 60.

FIG. 60.

*Molars:*—Supernumerary molars have only been found in the upper jaw. They occur sometimes in line in the normal arch, at other times protrude to the outer side, in which case they may cause trouble by wounding the cheek. They have also been found under the zygomatic process at the base of the ear, where they may cause an abscess which opens on the exterior, or have been known to protrude into the cranium.

*Diminution:*—Diminution in the number of the incisors is less frequent than the presence of supernumerary teeth. Diminution should not be confounded with cases of tardy eruption, nor with those cases of arrest of development, in which the teeth remain in their alveolar cavities, nor with cases of fracture or surgical injuries, and loss of teeth. Diminution includes only the complete abortion of the dental follicles, and can sometimes only be determined by examination of the jaw after death. Incisors, tush or molar teeth may be absent.

The tush tooth is the one which is most frequently absent.

[To be Continued.]

## Figures and Tables

**Fig. 56. f1:**
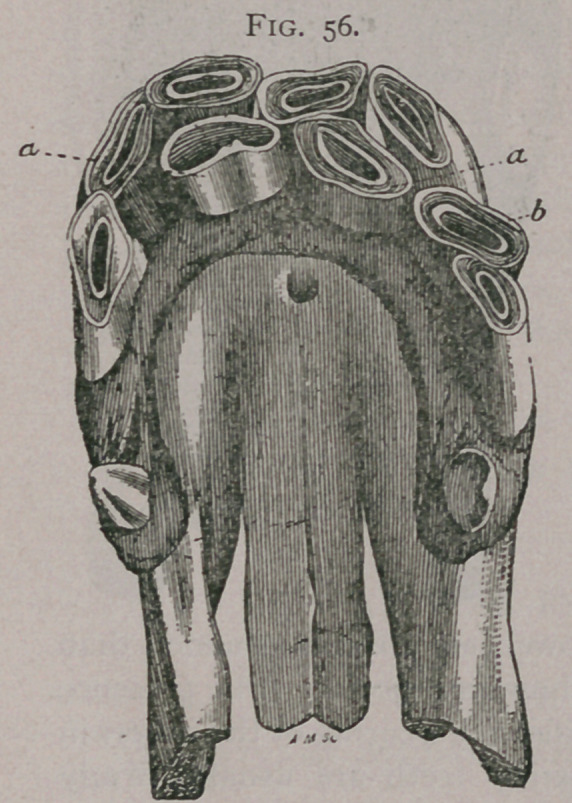


**Fig. 57. f2:**
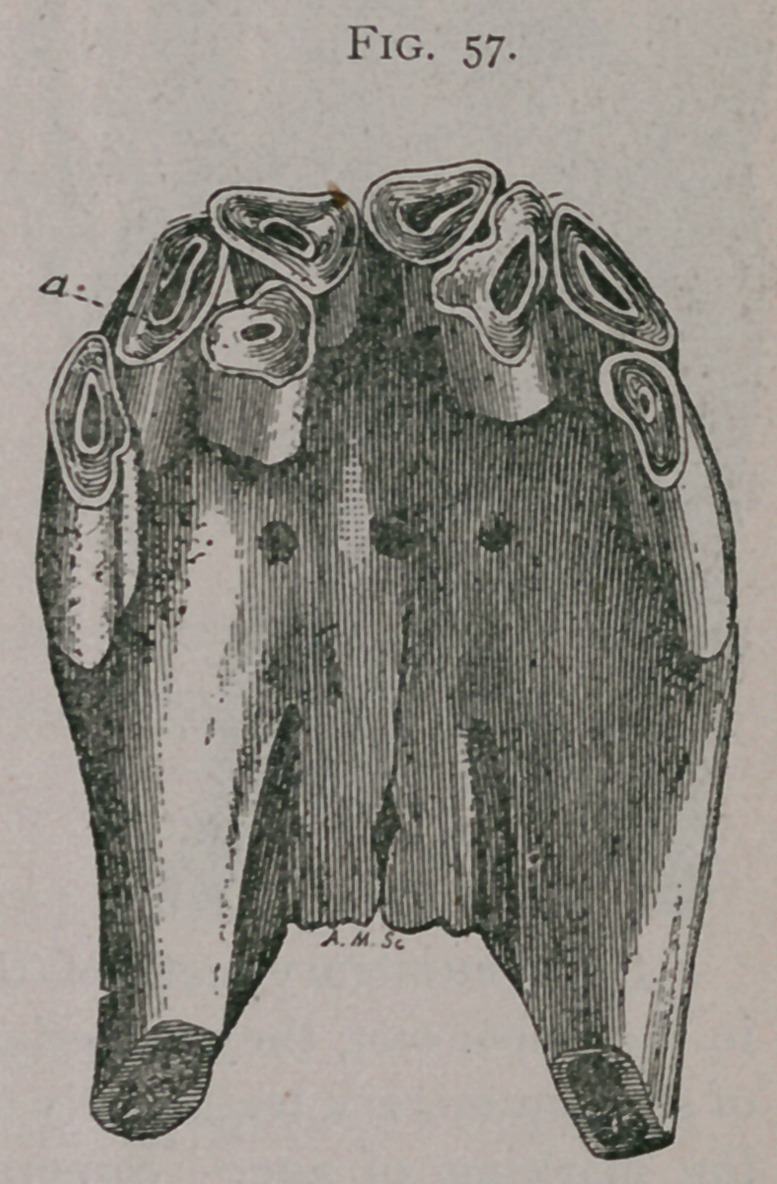


**Fig. 58. f3:**
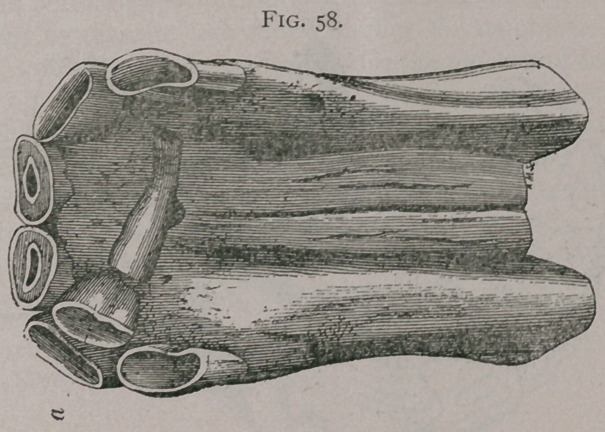


**Fig. 59. f4:**
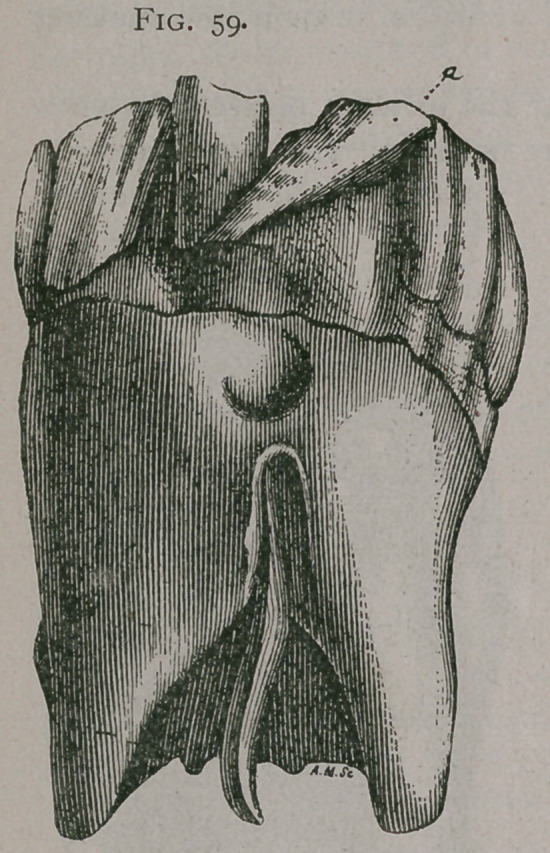


**Fig. 59a. f5:**
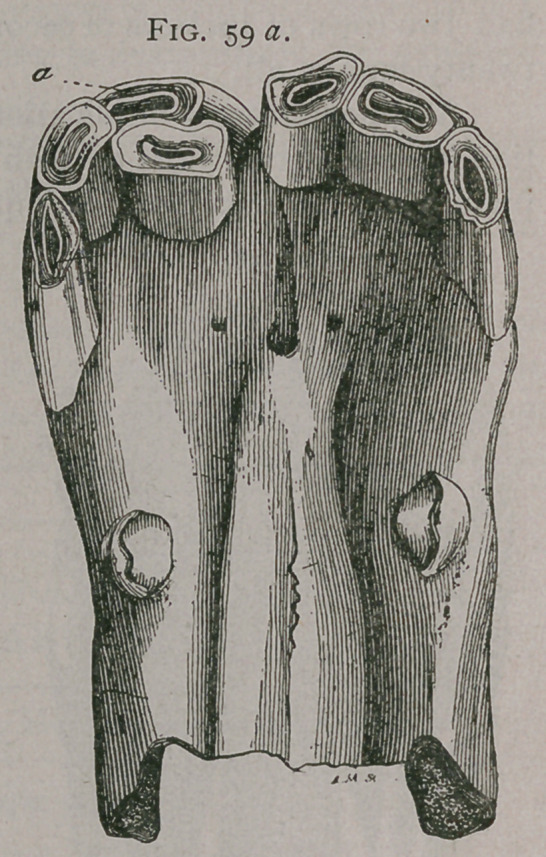


**Fig. 60. f6:**